# Y Incision Plus Anterior Extended Aortoplasty for Aortic Stenosis With Small Annulus and Sinus of Valsalva: The “Y and I” Incision Technique

**DOI:** 10.1016/j.atssr.2025.04.013

**Published:** 2025-05-12

**Authors:** Tadashi Kitamura, Yusuke Motoji, Masaomi Fukuzumi, Toshiaki Mishima, Ryoichi Kondo, Yoshimi Tamura, Saya Ishikawa, Kagami Miyaji

**Affiliations:** 1 Department of Cardiovascular Surgery, Kitasato University School of Medicine, Sagamihara, Japan

## Abstract

Aortic stenosis with a small annulus recently has been more frequently managed with the posterior annular enlargement technique. However, the risk of a short valve-to-coronary distance of the right coronary artery may remain in patients with small sinus of Valsalva. This report presents the case of a patient with severe bicuspid aortic stenosis with a small annulus and a small sinus of Valsalva who was treated with the “Y and I” incision technique, including posterior annular enlargement using the Y-incision technique and anterior extended aortoplasty.

The lifetime management concept is gaining greater attention in the treatment of aortic stenosis (AS), and valve-in-valve (ViV) transcatheter aortic valve implantation (TAVI) is currently an important option in lifetime management. Furthermore, the annular enlargement technique during surgical aortic valve replacement (SAVR) has recently been performed more frequently to address the future ViV TAVI to accommodate larger transcatheter valve prostheses. Posterior annular enlargement procedures, such as the Nicks,^1^ Manouguian,^2^ Nuñez,^3^ and Y-incision^4^ techniques, promote annular and posterior sinus of Valsalva enlargements. However, in patients with a small and short sinus of Valsalva, the anterior side of the sinus of Valsalva remains small and short, thus leaving concerns for short valve-to-coronary distance (VTC) and short valve-to-sinutubular junction distance (VTJ) in future ViV TAVI procedures. In this report, we present the “Y and I” incision technique, which allows posterior annular and sinus enlargement, as well as anterior sinus enlargement.

A 72-year-old woman with a history of hypertension presented with presyncope. Her body weight and body surface area were 41 kg and 1.3 m^2^, respectively. Echocardiography revealed a bicuspid aortic valve with severe AS, with a maximum transvalvular velocity, maximum pressure gradient, and mean pressure gradient of 5.8 m/s, 133.6 mm Hg, and 86.2 mm Hg, respectively. Computed tomographic angiography showed the following findings: height of the Valsalva sinus, 14.9 mm; aortic annular diameter, 16.4 mm × 23.0 mm; expected VTC of the right coronary artery after implanting a 21-mm valve prosthesis, 2.6 mm; diameter of sinus of Valsalva, 24.1 mm × 30.4 mm; and diameter of sinutubular junction, 24.1 mm × 25.7 mm (Figures 1A-1E). Implanting a 21-mm valve prosthesis with posterior annular enlargement alone was considered to result in residual short VTC and VTJ in the right coronary sinus postoperatively. Given the patient’s age, the Y and I incision technique was used, including posterior annular enlargement using Y-incision technique and anterior extended aortoplasty, on the basis of the possibility of future ViV TAVI.

**Figure 1:**
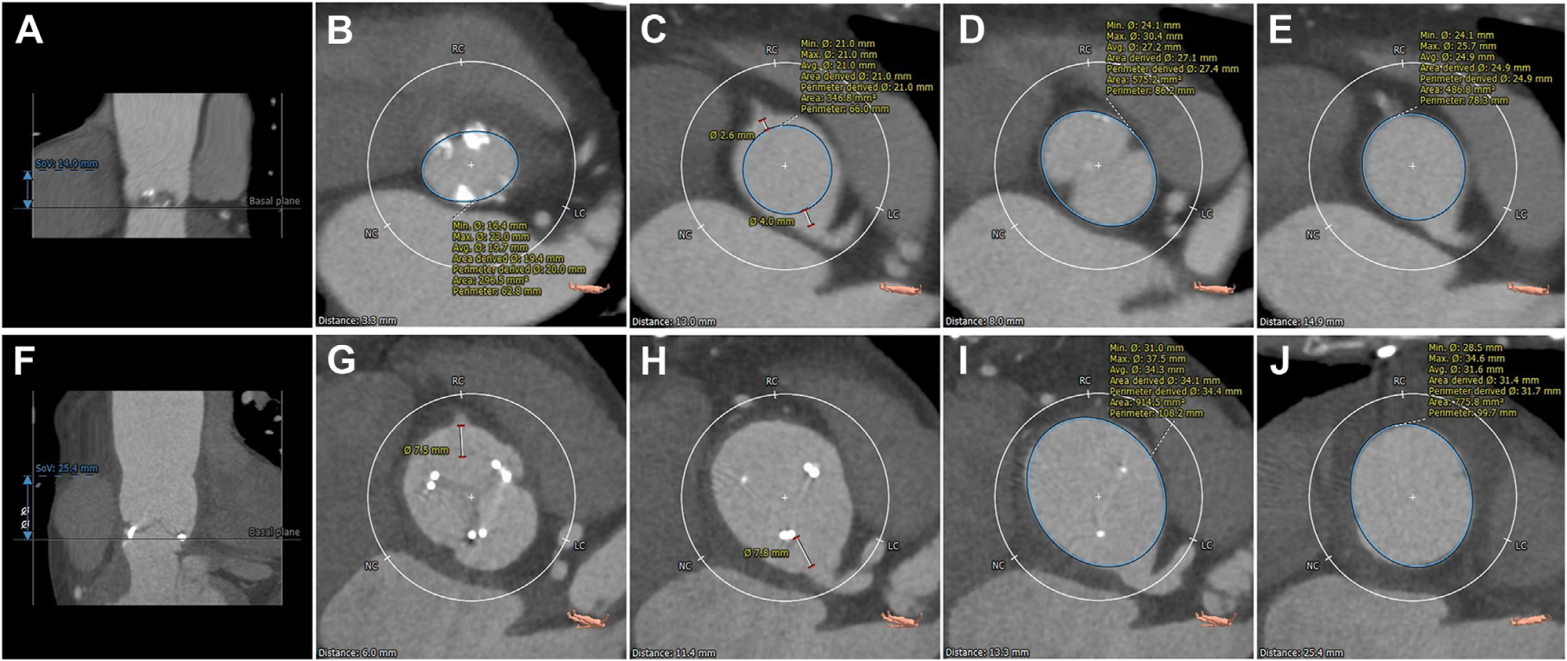
(A-E) Preoperative and (F-J) postoperative computed tomographic angiography. Preoperative images revealed that (A) the height of the sinus of Valsalva was 14.9 mm, (B) the aortic annulus was 16.4 mm × 23.0 mm, (C) the expected valve-to-coronary distance (VTC) of the right coronary artery after implanting a 21-mm valve prosthesis was 2.6 mm, (D) the diameter of the sinus of Valsalva was 24.1 mm × 30.4 mm, and (E) the diameter of the sinutubular junction was 24.1 mm × 25.7 mm. Postoperative images revealed that (F) the height of the sinus of Valsalva was 25.4 mm, (G) the VTC of the right coronary artery was 7.5 mm, (H) the VTC of the left coronary artery was 7.8 mm, (I) the diameter of the sinus of Valsalva was 31.0 mm × 37.5 mm, and (J) the diameter of the sinutubular junction was 28.5 mm × 34.6 mm. (Avg., average; Max., maximum; Min., minimum; LC, left coronary cusp; NC, noncoronary cusp; RC, right coronary cusp; SoV, sinus of Valsalva.)

## Technique

**Figure 2:**
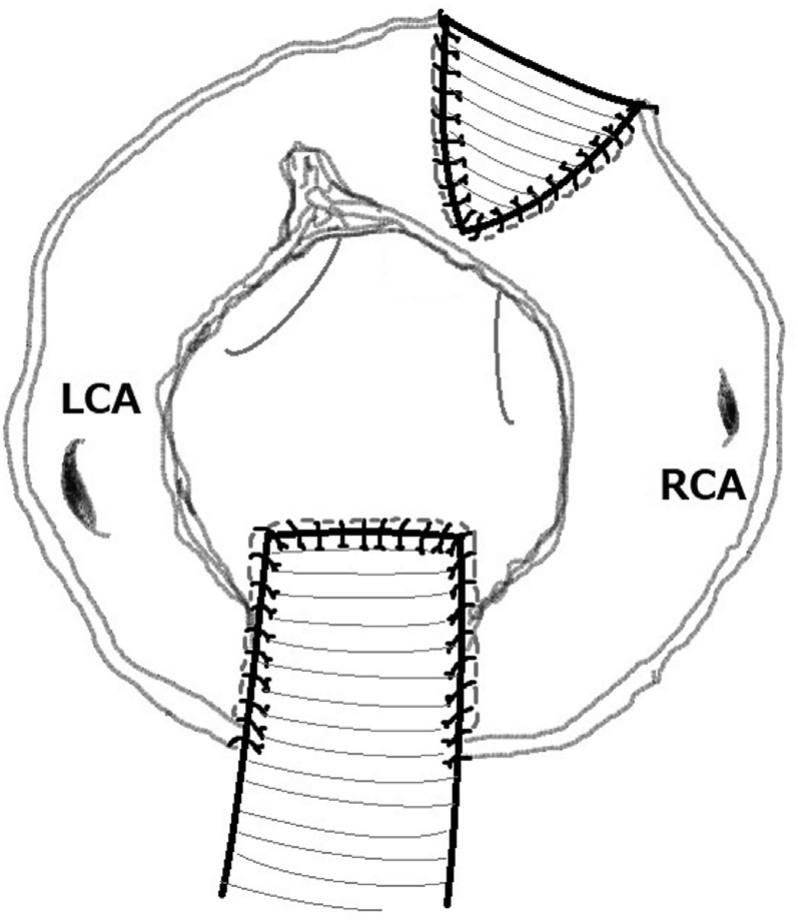
The “Y and I” incision technique. (LCA, left coronary artery; RCA, right coronary artery.)

Cardiopulmonary bypass was established using right atrial drainage and aortic perfusion. Cardiac arrest was obtained with antegrade and retrograde cardioplegia. The Sievers type 0 bicuspid aortic valve was excised through oblique aortotomy, and a cavitron ultrasonic surgical aspirator (Sonopet, Stryker Corp) was used to debride severe annular calcification thoroughly. The cylinder of the 19-mm valve sizer did not pass through the annulus. Aortotomy was extended beyond the posterior commissure to make a Y-incision underneath the ostium of left coronary artery on the left side and just short of the posterior trigone on the right side. A 25-mm-wide quadrangular woven polyester patch (Hemashield, Getinge) was sutured using a 5-0 polypropylene running suture. The right sinus of Valsalva was dissected from the right ventricular outflow tract, and a longitudinal incision was made between the anterior commissure and ostium of the right coronary artery toward the annulus. A 20-mm wedge-shaped Hemashield patch was sutured using a 5-0 polypropylene suture (Figure 2). Then, a 21-mm bioprosthesis (Inspiris, Edwards Lifesciences) was implanted using pledgeted mattress sutures on the native annulus and plain horizontal sutures on the patch using 2-0 braided polyester sutures. The aortotomy was closed using the posterior patch (Video). Postoperative echocardiography revealed that the maximum transvalvular velocity, maximum pressure gradient, and mean pressure gradient were 2.0 m/s, 16.2 mm Hg, and 10.2 mm Hg, respectively. Computed tomographic angiography showed the following findings: height of the new Valsalva sinus, 25.4 mm; VTC of the right coronary artery, 7.5 mm; VTC of the left coronary artery, 7.8 mm; diameter of the sinus of Valsalva, 31.0 mm × 37.5 mm; and diameter of the sinutubular junction, 28.5 mm × 34.6 mm (Figures 1F-1J). The patient was uneventfully discharged home and was in New York Heart Association functional class I at the time of the outpatient visit.

## Comment

With the aging of the population, the life expectancy of a 72-year-old Japanese woman was reported to be 18.24 years in 2023 according to the Ministry of Health, Labour, and Welfare of Japan.^5^ Therefore, a nonnegligible chance of future ViV TAVI exists in this generation of patients undergoing SAVR. For this reason, performing an annular enlargement procedure is recommended at the time of SAVR in younger patients to accommodate a larger prosthetic valve, in view of future ViV TAVI. However, the size of the anterior side of the sinus of Valsalva does not change after the posterior annular enlargement procedure. Truesdell and colleagues^6^ reported changes in aortic root dimensions after the Y-incision technique and demonstrated that the VTC of the right coronary artery did not significantly change. If the suture line of the valve prosthesis on the patch is brought distally to insert a larger prosthetic valve, the valve position may be tilted, and the postoperative VTC of the right coronary artery may become shorter. Short VTC and VTJ are known as risk factors in ViV TAVI,^7^ and hence, attempting to implant an oversized valve may result in serious coronary complications during future ViV TAVI.

Extended aortoplasty is a classic and simple technique frequently used to treat congenital supravalvular AS by using autologous pericardium.^8^ In our Y and I incision technique, posterior aortoplasty was performed using the Y incision, and anterior aortoplasty was performed with a separate I incision for a longer VTC of the right coronary artery. Patients with AS with a small annulus and sinus of Valsalva, as well as a lower takeoff of the right coronary artery, specifically patients with a moderate or longer life expectancy, will benefit from this additional I incision to the Y incision, thereby avoiding the risk of postoperative short VTC and VTJ without incising the ventricular septum.

## Supplementary Data

Supplementary Video Legend

Video Intraoperative video of the “Y and I” incision technique.2
