# Supplementary material for: Y Incision Plus Anterior Extended Aortoplasty for Aortic Stenosis With Small Annulus and Sinus of Valsalva: The “Y and I” Incision Technique
Source: Ann Thorac Surg Short Rep. 2025 May 12;3(4):1074–6. doi: 10.1016/j.atssr.2025.04.013 (PMC12712157; doi:10.1016/j.atssr.2025.04.013)
Supplement: Supplementary Video Legend [file mmc1.docx]

**SUPPLEMENTARY VIDEO LEGEND**

Video. Intraoperative video of the “Y and I” incision technique.
